# Regional and temporal trends in blood mercury concentrations and fish consumption in women of child bearing Age in the united states using NHANES data from 1999–2010

**DOI:** 10.1186/s12940-017-0218-4

**Published:** 2017-02-17

**Authors:** Leanne K. Cusack, Ellen Smit, Molly L. Kile, Anna K. Harding

**Affiliations:** School of Biological and Population Health Sciences, College of Public Health and Human Sciences, Oregon State University, Corvallis, Oregon USA

**Keywords:** Fish consumption, Methylmercury, NHANES, Blood, Coastal, Regional, Fish

## Abstract

**Background:**

The primary route of exposure to methylmercury (MeHg), a known developmental neurotoxicant, is from ingestion of seafood. Since 2004, women of reproductive age in the U.S. have been urged to eat fish and shellfish as part of a healthy diet while selecting species that contain lower levels MeHg. Yet few studies have examined trends in MeHg exposure and fish consumption over time in this group of women with respect to their geographical location in the U.S.

**Methods:**

Data from six consecutive cycles of the National Health and Nutrition Examination Survey (NHANES), 1999–2010 (*n* = 9597) were used to determine trends in blood mercury for women aged 16–49 residing in different regions in the US, and according to age, race/ethnicity, income level, and fish consumption using geographic variables.

**Results:**

Overall, mean blood mercury concentrations differed across survey cycles and mercury concentrations were lower in 2009–2010 compared to 1999–2000. There were regional patterns in fish consumption and blood Hg concentrations with women living in coastal regions having the highest fish consumption in the past 30 days and the highest blood Hg levels compared to women residing inland.

**Conclusions:**

On average, U.S. women of reproductive age were consuming more fish and blood mercury levels were lower in 2009–2010 compared to 1999–2000. However, efforts to encourage healthy fish consumption may need to be tailored to different regions in the U.S. given the observed spatial variability in blood mercury levels.

**Electronic supplementary material:**

The online version of this article (doi:10.1186/s12940-017-0218-4) contains supplementary material, which is available to authorized users.

## Background

The general population is exposed to methylmercury, a known neurotoxicant, primarily from fish consumption [1–6]. Methylmercury concentrations vary within and between fish species by more than 100-fold [7, 8]. For instance, the concentrations of mercury ranges from <0.003 ppm (ppm) for shellfish such as scallops and shrimp, to many ppm for high end predatory fish such as tuna, swordfish, and shark [9, 10]. Freshwater fish such as walleye and northern pike can also have high methylmercury [11, 12]. Consequently, an individual’s methylmercury exposure largely depends on the type and frequency of fish species consumed.

Fish advisories target women of childbearing age due to the greater sensitivity of the developing fetus to methyl mercury’s toxicity. Yet, communicating the risks and benefits of seafood consumption is challenging due to the need to reduce MeHg exposure while encouraging consumption of fish which are the primary source of omega 3-fatty acids in the diet [10]. In the U.S., the Food and Drug Administration (FDA) and the Environmental Protection Agency (U.S. EPA) have issued a joint advisory for pregnant women, women who may become pregnant, nursing mothers and young children. The advisory recommends avoiding specific types of fish that contain high levels of mercury, including Gulf tilefish, shark, swordfish, and king mackerel, as well as limiting the intake of albacore tuna to 6 oz per week [9]. At the same time, the advisory also states that fish is a healthy food due to its unique nutrient profile and that women of child bearing age should consume fish low in mercury up to twice a week. The most recent recommendations released by the Dietary Guidelines for Americans, 2015 (DGA, 2015) put more emphasis on the benefits rather than simply stating that women should consume up to two meals per week of seafood that is low in methylmercury. However, research has demonstrated that while consumers are aware of the methylmercury found in fish and the risks associated with this, they are unaware of any specific advice regarding fish consumption [13, 14]. While more research is needed to determine how to promote healthy seafood consumption, it is reasonable to be concerned that mercury advice might lead to reduced fish intake or inhibit needed increased consumption, but the evidence that this actually has happened is weak [15].

To determine if U.S. women of childbearing age are adopting the intended practices embodied in fish advisories and opting to consume fish species with lower mercury levels, it is necessary to evaluate databases that contain geographic-specific data, in combination with blood mercury data and fish consumption data. The current reference level of methylmercury in blood, set by the U.S. EPA, is 5.8 μg/L. The reference level is the equilibrium blood mercury level that is associated with a dietary intake of methylmercury at the current reference dose of 0.1 μg/kg-bw/day. This reference level defines the long-term average level of mercury in blood that was judged to be without appreciable risk when the reference dose was promulgated. However, recent research and a re-analysis of the data used to determine the reference dose has challenged the appropriateness of this reference level [7, 16]. Differences have also been found in maternal and cord blood mercury levels due to bio-concentration of methylmercury across the placenta [8, 10, 17–20]. Since more recent research strongly suggests that the current reference level may be the level of exposure at which adverse effects begin to be observed, the level of 3.5 μg/L suggested by previous researchers may be a more relevant benchmark for comparison until an updated reference dose is determined [8, 21].

This study examines the regional variation and temporal trends of fish consumption patterns with regards to blood mercury levels in women of childbearing age in the U.S. from 1999 to 2010. We hypothesized that type of fish being consumed and quantity of fish consumption and therefore total whole blood mercury levels would vary by region. Specifically, that those living in coastal areas would have higher fish consumption and higher blood mercury levels than non-coastal residents and that this would also vary by geographic location in the U.S.

## Methods

### Study population

NHANES is a continuous national survey that evaluates the health and nutritional status of the non-institutionalized US population conducted by the National Center for Health Statistics (NCHS). This study was limited to data from women of childbearing age (16–49 years of age) in six consecutive cycles of NHANES spanning from 1999 to 2010 (*N* = 9,597). In addition to the publically available data, this analysis used the respondent’s county as a geographic unit. This is a restricted variable and special permission to access this data was granted from the NCHS. Procedures for accessing restricted variables can be found online.

### Fish consumption data

Participants completed an interview that asked them to recall the number of times they ate 31 types of fish or shellfish in the previous 30 days. No data were collected on the amount of each species consumed. Frequency of fish and shellfish consumption across the 30-day recall period was calculated as total consumption and by type of fish consumed; *a*) tuna, *b*) predator fish (shark and swordfish), *c*) marine fish (fish sticks, haddock, mackerel, porgy, salmon, sardines, sea bass, unknown, other unknown, pollock and flatfish) *d*) freshwater fish (catfish, perch, pike, trout, bass and walleye) and *e*) marine shellfish (crab, crayfish, lobster, mussels, oyster, scallops, shrimp, other shellfish, unknown other shellfish).

### Blood mercury data

The NHANES data files contain data on total blood mercury (tHg) and blood inorganic mercury (iHg). The analytical method for measuring tHg in blood has been described in detail by the NCHS [12]. Briefly, tHg was measured using cold-vapor atomic absorption spectrophotometry with a detection limit of 0.14 μg/L. As 90–95% of mercury found in fish is methylmercury, tHg can be assumed to represent methylmercury [10]. Methylmercury in blood (MeHg) is calculated by subtracting iHg from tHg. Since the limit of detection (LOD) for iHg is larger than the LOD for tHg this approach may result in negative values. To address this problem, we followed the protocols described by Mahaffey et al. (2009) where MeHg = tHg - iHg if the difference is ≥ 0. If the difference is < 0, MeHg = 0.2 μg/L which is one half. If it is assumed that MeHg has the same LOD as iHg, then MeHg can be set equal to the LOD of iHg. We opted to conduct analysis using MeHg and tHg as the dependent variable [8]. Both models displayed similar trends and associations, thus we only report the results for total mercury. Of the 9,597 blood tHg measurements included in this analysis, 11% of the tHg measurements were below the limit of detection. Values below the level of detection limit were imputed by using a value equal to the detection limit divided by the square root of two.

### Geographical data

We hypothesized that fish consumption patterns would vary between residents who lived on or near the coast compared to those living inland. We also hypothesized that the type and quantity of fish consumed would vary by specific coast (ie., the types of fish consumed on the Pacific coast will be different than those consumed on the Gulf of Mexico). The participant’s county or county equivalent was used to categorize participants into four census regions and eight different regional groups: Atlantic Coast, Northeast, Great Lakes, Midwest, South, Gulf of Mexico, West and Pacific Coast. Additionally, we categorized participants as coastal or noncoastal. Any county that bordered the Pacific Ocean, Atlantic Ocean, the Gulf of Mexico, or the Great Lakes was considered coastal. Additionally, any county whose center point was within 25 miles of any coast was also considered coastal (See Additional file 1: Table S1 for a list of coastal counties) and was classified based on its proximity to the nearest largest water body.

### Covariates

Demographic data were included in the analysis as potential confounders including: race/ethnicity (Non-Hispanic White, Non-Hispanic Black, Other Hispanic, Mexican American and Other (which included Asians, Pacific Islanders, Native Americans and Alaska Natives), age (16–19, 20–29, 30–39 and 40–49 years of age), household income (<$20,000, $20,000-$44,999, $45,000-$74,999 and $75,000+), and survey cycle year.

### Statistical analysis

Population prevalence estimates for each census and coastal region were obtained for blood mercury levels ≥ 5.8 μg/L and ≥ 3.5 μg/L using appropriate sample weights, in order to determine the percentage of the population that has blood mercury levels greater than those are considered to be of concern for women of reproductive age. Blood mercury levels were natural log transformed to approximate a normal distribution. Bivariate linear regression models were used to examine the relationship between ln tHg (as a continuous variable) and cycle year, fish consumption, age, race/ethnicity, household income, type of fish and region of residence. Covariates that had an alpha >0.05 were then included in multivariate linear regression models.

Linear regression models were used to assess the relationship between tHg blood concentrations as a continuous variable and survey cycle year. Additional linear regression models were also used to evaluate the association between blood mercury concentrations and 30-day fish and shellfish consumption (total and by type of fish) controlling for race/ethnicity; income; time, geographical location and age. Temporal changes in tHg and fish consumption were evaluated using ANOVA and Tukey test.

Following NCHS guidance, the weights provided by NCHS were combined and weighted for all analyses reported in this study to account for the complex sampling design [12]. All analyses were performed using SAS, version 9.2 (SAS Institute Inc., Cary, NC).

## Results

**Fig. 1:**
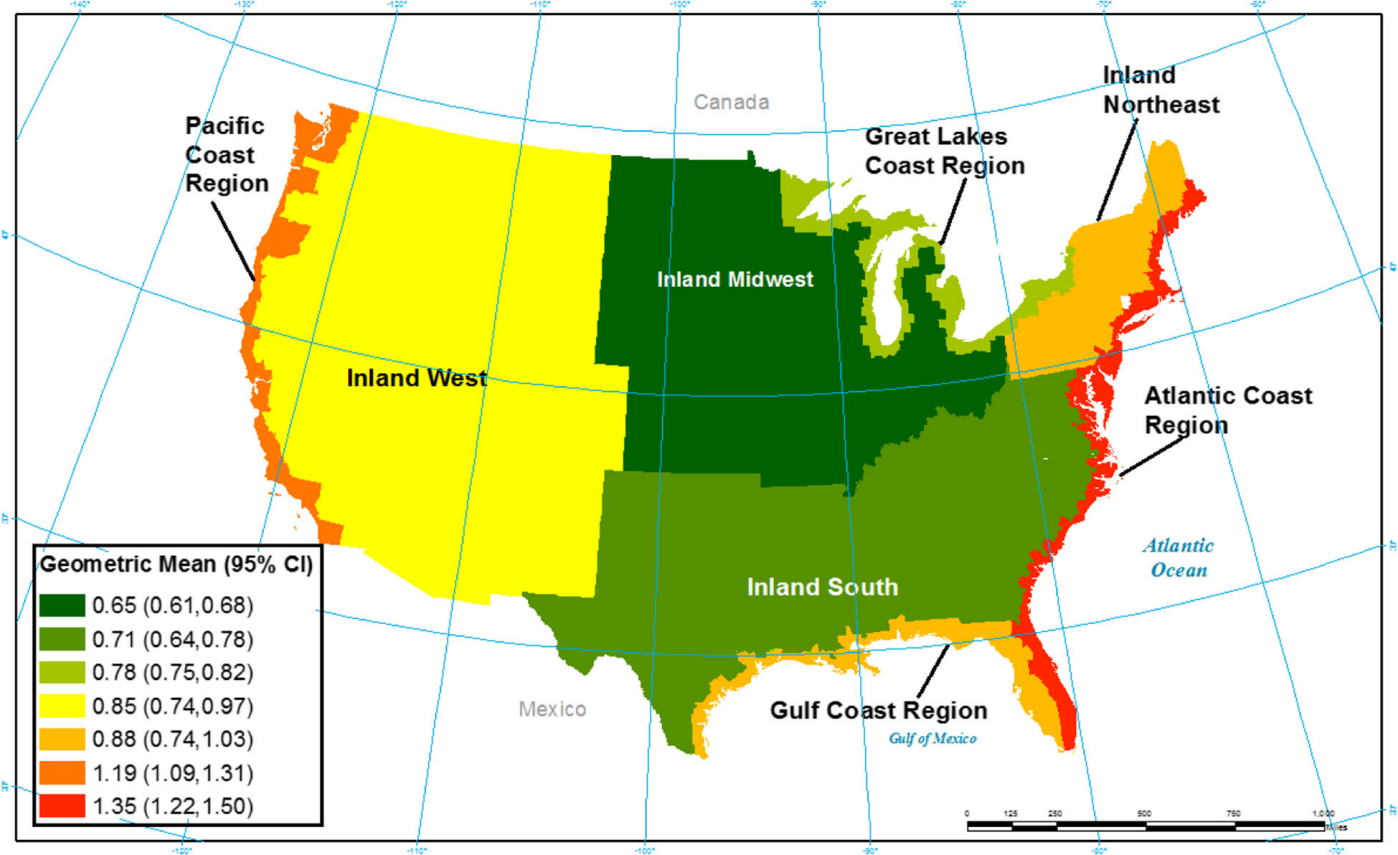
A map of whole blood mercury concentration (geometric mean and 95% Confidence Interval (μg/L)) in women of childbearing ages by coastal/inland regions for NHANES 1999–2010

Overall, blood mercury levels were observed to differ by region and coastal status in the US. The percentage of women by geographic census region and coastal status that had Hg concentrations ≥ 3.5 μg/L and ≥ 5.8 μg/L between 1999–2010 are presented in Table 1. In general, women in the Northeast had the highest percentage of blood Hg concentrations with 6.48% ≥ 5.8 μg/L and 15.21% ≥ 3.5 μg/L while women in the Midwest had the lowest percentages (0.78 and 2.64% respectively). Additionally, when blood Hg was evaluated as a continuous variable, mean blood Hg concentrations were also observed to differ by region and coastal status for U.S. women of childbearing age across all survey cycles (Fig. 1; Additional file 1: Table S1). Overall, women living in coastal regions had higher geometric mean Hg levels (1.12 μg/L; 95% CI 1.05 μg/L,1.20 μg/L) compared to those living in non-coastal areas (0.74 μg/L; 95% CI 0.70 μg/L −0.78 μg/L). Additionally, women living in the Atlantic and Pacific coastal region had the highest geometric mean Hg concentration of 2.41 μg/L, (95% CI 2.13 μg/L – 2.69 μg/L) and 1.97 μg/L, (95% CI 1.75 μg/L −2.19 μg/L), respectively. Women in the inland Midwest had the lowest geometric mean Hg concentrations of 0.94 μg/L (95% CI 0.88 μg/L −1.01 μg/L).

**Table 1:** Total percentage of women of childbearing ages (16–49 years) with mercury concentrations ≥ 3.5 μg/L and ≥ 5.8 μg/L by U.S. Census region and coastal status for NHANES 1999–2010, weighted (*N* = 9,597)

	U.S. Census Region			Pr > F	Coastal Status		Pr > F
Whole blood mercury	Northeast	South	Midwest	West		Coastal	Noncoastal
Percentage ≥ 3.5 μg/L 15.21	10.77	2.64	8.36	<0.001	12.01	4.37	<0.001
Percentage ≥ 5.8 μg/L 6.48	3.26	0.78	4.24	<0.001	6.06	1.81	<0.001

**Fig. 2:**
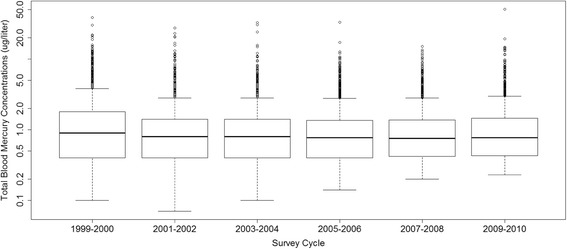
Distribution of Total Blood Mercury (ug/L), by NHANES survey cycle, for women of childbearing age

There was a statistically significant decrease in the mean blood mercury concentrations in 2009–2010 as compared to 1999–2000 (Fig. 2 and Additional file 1: Table S2). While there was no apparent decreasing trend over time, mean tHg levels in 1999–2000 were 1.96 ug/L (95% CI: 1.50, 2.42) compared to 1.39 ug/L L (95% CI: 1.26, 1.53).

**Fig. 3:**
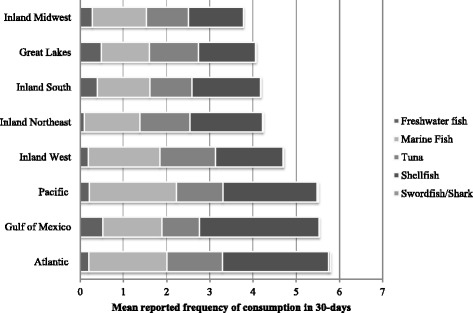
Mean reported fish consumption by species in NHANES participant women aged 16–49 years, by region for all years combined

Fish consumption was the most frequent in the Atlantic, Gulf of Mexico, and the Pacific regions (see Fig. 3 and Additional file 1: Table S3). Fish species consumed also varied by region. In all regions except the Inland West and Inland Midwest, shellfish was the most commonly consumed item. Freshwater fish was most often consumed by women living in the Gulf of Mexico coastal region and the least often consumed in the Inland Northeast. Marine fish was most often consumed by women living in the Pacific Coast and the least often by women in the Gulf of Mexico region. Tuna consumption was fairly similar in the Great Lakes, Inland Midwest and Inland Northeast and the lowest consumption was found in the Gulf of Mexico. Shellfish was consumed in the greatest frequency in the Gulf of Mexico and consumed the least in the Great Lakes. Swordfish and shark were consumed by less than 1% of all women in all regions (Additional file 1: Table S2).

**Fig. 4:**
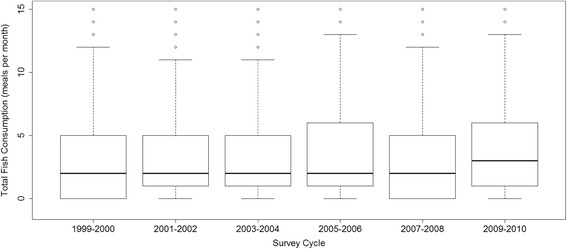
Distribution of total fish consumption (meals per month), by NHANES survey cycle, for women of childbearing age

The mean weighted frequency of fish consumption in the past 30 days for each survey cycle is displayed in Additional file 1: Table S2. The total number of times fish was consumed by US women of childbearing age differed across the six survey cycles (*p* = 0.05). Compared to 1999–2000, U.S. women consumed on average one additional fish meal per 30 days in the survey cycle 2009–2010 (Fig. 4 and Additional file 1: Table S2). The percentage of U.S. women who reported not eating any fish in the past month in the 2009–2010 cycle has decreased since 1999–2000 (23% compared to 26%). While there was no consistent trend over time, this data indicated that U.S. women of reproductive age, on average, were consuming more fish per month in 2009–2010 compared to 1999–2000. The data also showed that the frequency of marine fish consumption (*p* = 0.01) and shellfish consumption (*p* = 0.02) had increased slightly each year since 1999 with the exception of 2007–2008. Yet there was no appreciable difference in the mean number of times freshwater fish (p = 0.24), tuna (*p* = 0.09) or predatory fish (*p* = 0.55) were consumed over this time frame.

**Fig. 5:**
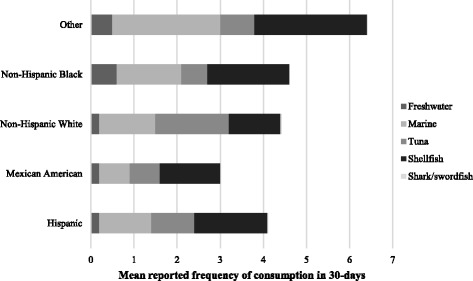
Mean reported fish consumption by species in NHANES participant women aged 16–49 years, by race/ethnicity for all years combined

Age, income, and race/ethnicity were associated with higher fish consumption in bivariate analysis. Specifically, as age increased so did total fish consumption (β 0.08, 95% CI: 0.06,0.09) (Additional file 1: Table S3). Age was also associated with an increase in consumption of marine fish (*p* < 0.01), freshwater fish (*p* < 0.01), tuna (*p* < 0.01) and shellfish (*p* < 0.01). A categorical increase in total household income was associated with a statistically significant increased total fish consumption (*p* < 0.01), marine fish (*p* < 0.01), tuna (*p* < 0.01), shellfish (*p* < 0.01), freshwater fish (*p* = 0.02) and swordfish/shark (*p* = 0.05). Finally, participants who self-identified as ‘Other’ (Asian-Americans, Pacific Islanders, Alaska Natives and Native Americans) consumed the greatest amount of total fish per month (6.4 ± 8.8) and Mexican Americans were consuming the least fish per month (3.0 ± 4.1). The ‘Other’ category consumed the greatest amount of marine fish in the last 30-days and Mexican Americans consumed the least amount. Freshwater fish was consumed the most by Non-Hispanic Blacks in the last 30 days and consumed the least by Hispanics. Tuna was consumed the most frequently by Non-Hispanic whites and by Non-Hispanic Blacks the least. Swordfish/shark was consumed the most by frequently by Non-Hispanic whites and by Non-Hispanic Blacks the least. Shellfish was consumed the most frequently by the ‘Other’ category and the least by Mexican Americans (see Fig. 5, Additional file 1: Table S2).

Blood Hg concentrations were associated with income, age, race/ethnicity, total fish, and region. Blood Hg concentrations increased with increasing age and income, with those with a household income of ≥ $75,000 having higher Hg concentrations (β = 0.43, 95% CI: 0.26,0.61) than those reporting a household income less than $20,000. Mexican Americans had the lowest Hg concentrations (β = −0.09, 95% CI: −0.21,0.02) compared to Non-Hispanic Whites. On average, people who self-identified as ‘Other’ race had more Hg in their blood (β = 1.03, 95% CI: 0.65,1.40) compared to those in the Non-Hispanic White category. Blood mercury was higher in women residing in the Atlantic Coast (β = 0.47, 95% CI: 0.08,0.86) and the Pacific coast (β = 0.22, 95% CI: −0.18,0.63) compared to women in the inland South after controlling for fish consumption, age, race-ethnicity, income, and survey cycle. Conversely, blood mercury levels were lower in women residing in the Gulf Coast (β = −0.36, 95% CI: −0.76,–0.04), the Great Lakes Coast (β = −0.54, 95% CI: −0.88,–0.19), the Inland Midwest (β = −0.58, 95% CI: −0.93,–0.23), Inland West (β = −0.30, 95% CI: −0.93,–0.23) and Inland Northeast (β = −0.43, 95% CI: −0.77,–0.09), compared to Inland South in adjusted models while controlling for the same covariates (Table 2).

**Table 2:** Multiple linear regression model describing the associations between total blood mercury levels adjusted for other covariates among women 16–49 years of age participating in NHANES during 1999–2010

	Model 1^a^	Model 1^b^	Model 1^c^
	β (95% CI)	β (95% CI)	β (95% CI)
Intercept	0.81(0.45,1.17)	0.61(0.32,0.90)	0.48(0.23,0.72)
Survey Cycle			
1999–2000	0.0 (ref)	0.0 (ref)	0.0 (ref)
2001–2002	–0.44(–0.72,–0.17)*	–0.57(–0.95,–0.19)*	–0.47(–0.79,–0.14)*
2003–2004	–0.53(–0.78,–0.27)*	–0.58(–0.95,–0.22)*	–0.67(–0.97,–0.37)*
2005–2006	–0.56(–0.88,–0.24)*	–0.59(–0.97,–0.21)*	–0.57(–0.91,–0.22)*
2007–2008	–0.57(–0.82,–0.31)*	–0.73(–1.09,–0.38)*	–0.66(–0.96,–0.35)*
2009–2010	–0.68(–0.93,–0.43)*	–0.67(–1.03,–0.31)*	–0.71(–1.00,–0.41)*
Income			
<$20,000	0.0 (ref)	0.0 (ref)	0.0 (ref)
$20,000–$44,999	0.12(–0.02,0.26)	0.15(0.02,0.28)*	0.12(–0.02,0.26)
$45,000–$74,999	0.13(0.00,0.26)*	0.17(0.04,0.31)*	0.11(–0.02,0.24)
$75,000+	0.43(0.26,0.61)*	0.54(0.36,0.71)*	0.41(0.24,0.59)*
Race/Ethnicity			
Non-Hispanic White	0.0 (ref)	0.0 (ref)	0.0 (ref)
Mexican American	–0.09(–0.21,0.02)	–0.04(–0.16,0.08)	–0.11(–0.22,0.01)
Other Hispanic	0.0 (–0.31,0.32)	0.35(–0.02,0.72)	0.15(–0.18,0.48)
Other	1.03(0.65,1.40)*	1.17(0.80,1.55)*	1.03(0.66,1.39)*
Non-Hispanic Black	0.11(0.00,0.22)*	0.25(0.13,0.38)*	0.09(–0.02,0.21)
Age			
16–19	0.0 (ref)	0.0 (ref)	0.0 (ref)
20–29	0.19(0.10,0.29)*	0.26(0.17,0.35)*	0.19(0.09,0.28)*
30–39	0.41(0.23,0.59)*	0.46(0.28,0.65)*	0.41(0.22,0.59)*
40–49	0.37(0.25,0.48)*	0.44(0.34,0.55)*	0.35(0.24,0.47)*
Fish Consumption/month			
0	0.0 (ref)	0.0 (ref)	0.0 (ref)
1–4	0.52(0.41,0.63)*	–	0.52(0.41,0.62)*
5–8	1.12(0.99,1.24)*	–	1.14(1.01,1.27)*
9+	1.97(1.78,2.17)*	–	2.02(1.81,2.22)*
Region			
Inland South	0.0 (ref)	–	–
Atlantic Coast	0.47(0.08,0.86)*	–	–
Gulf Coast	0.36(–0.76,0.04)	–	–
Pacific Coast	0.22(–0.18,0.63)	–	–
Great Lakes Coast	–0.54(–0.88,–0.19)*	–	–
Inland West	–0.30(–0.69,0.08)	–	–
Inland Midwest	–0.58(–0.93,–0.23)*	–	–
Inland Northeast	–0.43(–0.77,–0.09)*	–	–
Type of fish			
Swordfish and Shark	–	1.80(0.57,3.01)*	–
Tuna	–	0.13(0.09,0.17)*	–
Shellfish	–	0.09(0.06,0.12)*	–
Marine fish	–	0.08(0.01,0.15)*	–
Freshwater fish	–	0.12(0.02,0.21)*	–
Coastal Status			
Non-coastal	–	–	0.0 (ref)
Coastal Status	–	–	0.50(0.33,0.67)*

^a^Multiple linear regression model adjusted for survey cycle, household income, age, race/ethnicity, fish consumption and region of residence

^b^model adjusted for survey cycle, household income, age, race/ethnicity, fish consumption, type of fish

^c^model adjusted for survey cycle, household income, age, race/ethnicity, fish consumption and coastal status

*p < 0.05

## Discussion

The current study observed that U.S. women of childbearing age who live in coastal regions consumed more fish per month and had higher whole blood Hg concentrations compared to women living in the Midwest after controlling for other confounders. In particular, women who lived in the Atlantic or Pacific coastal regions had the highest fish intake and the highest blood Hg concentrations. These results are consistent with other studies which have observed differences in mercury exposure even within a single state due to location of residence (coastal/non-coastal), type of fish consumed, and consumption rates [1, 22–24]. Compared to the results of a previous study by Mahaffey et al. (2009), who examined women of childbearing age using NHANES data from 1999–2004, we saw a modest decrease in the geometric mean blood mercury concentrations for women residing in the Atlantic coast, from 1.55 μg/L to 1.35 μg/L, and the Gulf of Mexico, from 0.96 μg/L to 0.88 μg/L, but a modest increase for women residing in the Inland Northeast from 0.77 μg/L to 0.85 μg/L and no change in other regions when adding in the additional 2005–2010 NHANES survey cycles [10].

Women living in coastal areas were at greater risk of having blood mercury concentrations higher than the 5.8 μg/L reference level (6.1 vs 1.8% for non-coastal areas). Women living in the Northeast were at the greatest risk for having blood mercury concentrations greater than 5.8 μg/L (6.5%), compared to the other census regions. The reference level is the equilibrium blood mercury level that is associated with a dietary intake of methylmercury at the current reference dose of 0.1 μg/kg-bw/day. This reference level defines the long-term average level of mercury in blood that was judged to be without appreciable risk. However, recent research and a re-analysis of the data used to determine the reference dose (0.1 μg/kg-bw/day) has challenged the appropriateness of this reference level [7, 16]. Since more recent research strongly suggests that the current reference level may be the level of exposure at which adverse effects begin to be observed, the amended level of 3.5 μg/L suggested by previous researchers may be a more relevant benchmark for comparison until an updated reference dose is determined. Using 3.5 μg/L, we saw similar, yet more pronounced, patterns. Women in the Northeast were still at the greatest risk, with 15.21% greater than the suggested reference level. Women living in coastal regions were still at greater risk of having blood mercury concentrations greater than the suggested 3.5 μg/L level compared to the non-coastal areas.

Importantly, we also observed that total monthly fish consumption by U.S. women of reproductive age was higher in recent years. Specifically, in 2009–2010 marine and shellfish consumption had increased by approximately one additional fish meal per month compared to 1999–2000 yet consumption of freshwater fish, tuna and swordfish/shark had decreased slightly over time. This is encouraging considering that the consumption of marine and shellfish was associated with the smallest increase in total whole blood mercury 0.08 (95%CI: 0.01,0.15) and 0.09 (95%CI: 0.06,0.12), respectively. Notably, there was also a statistically significant decrease in mean whole blood mercury levels between 1999–2000 and 2009–2010.

On average women who ate fish at a greater frequency (9+ times in the past month) in 2009–2010 had lower blood mercury levels than women who ate fish at the same rate in 1999–2000. Women who ate fish 9+ times in the past 30 days in 2009–2010 had an arithmetic mean blood mercury level of 2.4 μg/L (95%CI: 2.1,2.7), compared to 4.1 μg/L (95%CI: 3.5,4.7), in 1999–2000 in a bivariate analysis.

The observed increase in fish consumption and corresponding decrease in blood mercury levels may be attributed to several different possibilities. In the most recent survey cycle (2009–2010) 34% of the fish consumption was from marine fish, 18% from tuna, 42% from shellfish, 5% from fresh water fish and lest than a quarter percent from swordfish or shark. Swordfish and shark have the strongest association with increase in blood mercury levels (β1.80, 95%CI: 0.57,3.01) followed by tuna and freshwater fish. However they account for such a small percentage of the fish being consumed in the U.S., it seems unlikely that the consumption of these species is affecting blood mercury levels nationally. The decline in women's blood mercury levels in the NHANES samples may have been driven largely or in part by market changes; for example, over the decade studied, market shares for low-mercury varieties including shrimp, tilapia, salmon and catfish have increased dramatically, while shares of high-mercury varieties were decreasing, as did consumption of those high-mercury fish by women of childbearing age, as already noted. Tuna is of particular interest, since in 2014 it accounted for 14% of the US seafood market (FDA 2014). It is therefore plausible that differences in consumption of tuna fish among regions or age or ethnic groups might be associated with differences in blood mercury levels. If so, that would have major implications for seafood consumption advice.

Consistent with other studies, we found that as age and income increases, fish consumption was increasing [25, 26]. However, the 40–49 years women had lower blood mercury concentrations than the 30–39 years olds. Older women (40–49 years) are also consuming more total fish (5.1 meals/month) compared to younger women (15–19 years: 2.6 meals/month) and consuming more swordfish/shark and freshwater fish as compared to the younger age categories. As fish consumption advisories are typically aimed at women of childbearing age, it is possible that older women, who no longer plan on bearing children, may not pay heed to fish consumption advice if they feel the advice no longer pertains to them. Advice still needs to focus on encouraging younger women to consume more fish that is low in mercury and in high omega 3 as 36.8% of the women aged 16–19 and 24.1% of women aged 20–29 were consuming no fish at all.

We also found that, similar to previous studies, those who identified as Alaska Native/American Indian, Pacific Islander, Caribbean Islander or Asian (“Other” race/ethnicity” category) consumed the most fish per month and had the highest blood mercury levels, but 80% of the reported fish consumption was from marine or shellfish [26]. Mexican Americans ate the least amount of fish per month and had the lowest blood mercury levels. 70% of the fish they consumed was marine or shellfish. Looking at geographic differences in fish consumption, the Atlantic coast was consuming the most total fish per month with the majority being marine and shellfish. An increase in household income was also associated with greater fish consumption as well as blood mercury levels. Geographic differences may occur as a result of fresh fish being more available in coastal areas and because the cuisine in coastal areas emphasizes fish more so than inland cuisine [22].

Geographic-specific and demographic data are important in order to develop meaningful fish consumption advisories. Fish consumption advisories are frequently based on nationally aggregated estimates of methylmercury concentrations found in fish as well as fish consumption rates representing a specific population, such as women of child bearing age [25]. Determining the site-specific contributions to mercury in the environment, as well as the specific fish being consumed by the local population would be beneficial particularly in an area such as the northeastern part of the US and along the Atlantic coast, where there are both higher blood mercury concentrations and fish consumption. Specific demographic groups are also more susceptible to mercury toxicity due to age, race/ethnicity, cultural identity and practices, and coastal proximity [8, 27–30]. Fish consumption advisories need to be tailored to reflect the fish consumption habits of the population at being targeted or for those at risk.

Research has demonstrated that fish consumption advisories are often not reported effectively. Recent studies have found that awareness of advisories was lowest among women and in particular pregnant women, non-White ethnic groups, senior citizens, people age 15–19, low income and people with less than a high school education [22, 28, 31]. Researchers in California found that awareness of the advisory does not guarantee that the information provided is understood or accepted [31]. The targeted audience may disregard the advice for several reasons; the advice may contradict long held cultural beliefs, or they may be unconcerned about the potential health effects, or find that the evidence for harm is lacking [31–37]. Often times the fish consumption advisories focus on the fish species that are the highest risk for methylmercury exposure and do not include examples of species which are low in methylmercury and high in polyunsaturated fats. This method undermines the health and nutritional benefits of fish consumption and in turn, women of reproductive age may be lacking the nutritional benefits garnered from fish. The information needs to be presented in a balanced way to enhance the acceptance and to prevent complete avoidance of such an important food group.

Often times State agencies issue lengthy and detailed advisories that have been difficult for ethnically diverse groups and non-English speakers to interpret [31, 38, 39]. Advice that is appropriate for Asians may not be applicable to Native Americans, or, species and quantities consumed by Koreans may be very different from those consumed by Vietnamese people. The ‘Other’ category in the NHANES survey, includes a diverse mix of racial groups. When crafting fish consumption advisories, care must be taken to ensure that the information is appropriate for the varied fish consumption preferences and habits of these subgroups.

Despite the general increased trend in fish consumption, only 21% of U.S. women in 2009–2010 consumed fish at a rate of twice a week (8–12 oz/week) as recommended by the most recently updated Dietary Guideline for Americans (DGA, 2015). The updated guidelines urge women of childbearing age to eat 8–12 oz of fish per week and provides a list of nine seafood varieties that are both high in omega-3 s and low in mercury (DGA, 2015). While this percentage is significantly higher than 1999–2000 in which only 12% of the population was consuming fish at the recommended rate, women of reproductive age may be lacking the nutritional benefits garnered from fish. Lando et al. found similar results in which nearly all women in their study consumed much less than the current recommendations and pregnant and postpartum women may not be eating enough low mercury fish in order to gain the benefits of fish consumption [22]. Ways need to be found to encourage all women to consume more fish that is low in mercury and high in omega three fatty acids. Fish are an excellent source of high quality protein, they contain vitamins and other essential nutrients, as well as high levels of omega-3 poly unsaturated fats [40]. Improvement in fetal outcomes such as longer gestation, increased birth weight and benefits in fetal brain development has been reported for women who consume fish during pregnancy [1, 41].

As discussed by Groth, fish consumption advisories need to clearly delineate which fish species can be consumed often, or conversely, should be avoided [7]. Rather than solely focusing on species to avoid, it would be useful to provide a broad range of fish that are low in methylmercury and high in omega 3 s as well. This list should be comprehensive to not only include fish that may be consumed by a range of different ethnic groups, but fish that may also be specific to geographic regions. An improvement in the reporting of fish consumption advisories is necessary and could be achieved by providing informational material to health clinics, pediatricians, and gynecologists in order to reach the high risk populations [28].

While our study has many strengths including the use of 6 cycles of NHANES data that collected questionnaire data and biomarker data using the same methods across each cycle coupled with the restricted geographical data, there were also limitations that are worth noting. For instance, we used a 30-day food recall questionnaire to ascertain fish intake. This length of recall could introduce misclassification and has potential for bias. However, this questionnaire was used consistently across all cycles and consequently should be internally valid since it is unlikely that the recall bias that is inherent in food frequency questionnaires would differ from one survey cycle to the next. In addition, fish and shellfish are generally easily identifiable foods and therefore more readily recalled than other food groups [8]. Additionally, the validity of the dietary recall for fish consumption has been found to be greater than for all other food groups [42, 43]. However, the questionnaire only collected data on frequency and thus we do not know amounts consumed nor were we able to determine if portion sizes changed over time.

## Conclusion

Fish advisories are tasked with balancing the benefits of fish consumption with reducing the risk of mercury exposure. While it appears that whole blood mercury levels are decreasing and fish consumption is increasing over time, a substantial number of women of reproductive age in the U.S. still have blood mercury levels that are above those recommended by the EPA’s current reference level and even more are above the suggested level of 3.5 μg/L. A large portion are not eating fish twice a week as recommended by the Dietary Guidelines for Americans. Risk managers and physicians need to consider the target demographics for fish consumption advisories, how populations will respond to fish these advisories, how those responses will influence nutrient intake and methylmercury exposure, and the affect this will in turn have on public health [44]. An emphasis needs to be on providing women of reproductive ages with advice that highlights the positive benefits of fish consumption, particularly during pregnancy, and provides examples of fish that are low in mercury and high in omega 3 s, rather than simply pointing out the fish that are high in mercury. The advisories need to be broad to include fish consumed by a range of ethnically diverse populations, and as region-specific as possible.

## Additional file

Additional file 1: Table S1. Distribution of blood total mercury (μg/L), women 16–49 years of age, by region, coastal status and survey cycle NHANES 1999–2010. **Table S2.** Difference in mean blood mercury levels, for women 16–49 years of age, by survey cycle NHANES 1999–2010. **Table S3.** Total fish consumption and type of fish consumed, by selected demographic variables among women 16–49 years of age participating in NHANES during 1999–2010. (DOCX 29 kb)

## Abbreviations

AMA: American Medical Association
DGA: Dietary Guideline for Americans
FDA: Food and Drug Administration
iHg: Blood inorganic mercury
MeHg: Methylmercury
NCHS: National Center for Health Statistics
NHANES: National Health and Nutrition Examination Survey
tHg: Total blood mercury
U.S. EPA: United States Environmental Protection Agency
