# Supplementary material for: Regional and temporal trends in blood mercury concentrations and fish consumption in women of child bearing Age in the united states using NHANES data from 1999–2010
Source: Environ Health. 2017 Feb 17;16:10. doi: 10.1186/s12940-017-0218-4 (PMC5316155; doi:10.1186/s12940-017-0218-4)
Supplement: Additional file 1: Table S1. — Distribution of blood total mercury (μg/L), women 16–49 years of age, by region, coastal status and survey cycle NHANES 1999–2010. Table S2. Difference in mean blood mercury levels, for women 16–49 years of age, by survey cycle NHANES 1999–2010. Table S3. Total fish consumption and type of fish consumed, by selected demographic variables among women 16–49 years of age participating in NHANES during 1999–2010. (DOCX 29 kb) [file 12940_2017_218_MOESM1_ESM.docx]

Table 1. Distribution of blood total mercury (µg/L), women 16-49 years of age, by region, coastal status and survey cycle NHANES 1999-2010.

|  | **N** | **Arith. Mean** | | **Geometric Mean** | |  |  | **Selected percentiles (95% CI)** | | |  |  |  |
| --- | --- | --- | --- | --- | --- | --- | --- | --- | --- | --- | --- | --- | --- |
|  |  | **(95% CI)** | | **(95% CI)** | |  |  |  |  |  |  |  |  |
| **Region** | |  |  |  |  | **25th** |  | **50th** |  | **75th** |  | **90th** |  |
| **Atlantic coast** | 1662 | 2.41 | (2.13,2.69) | 1.35 | (1.22,1.50) | 0.64 | (0.57,0.72) | 1.37 | (1.22,1.53) | 2.89 | (2.47,3.38) | 5.36 | (4.65,6.18) |
| **Gulf of Mexico** | 541 | 1.41 | (0.42,2.41) | 0.88 | (0.49,1.59) | 0.49 | (0.26,0.95) | 0.83 | (0.48,1.43) | 1.6 | (1.02,2.49) | 2.99 | (2.54,3.61) |
| **Pacific Coast** | 1566 | 1.97 | (1.75,2.19) | 1.19 | (1.09,1.31) | 0.59 | (0.54,0.65) | 1.2 | (1.05,1.36) | 2.38 | (2.07,2.73) | 4.47 | (3.77,5.31) |
| **Great Lakes** | 708 | 1.09 | (1.04,1.13) | 0.78 | (0.75,0.82) | 0.46 | (0.41,0.51) | 0.82 | (0.78,0.85) | 1.39 | (1.31,1.48) | 2.13 | (1.83,2.48) |
| **Inland West** | 1237 | 1.33 | (1.14,1.52) | 0.85 | (0.74,0.97) | 0.45 | (0.4,0.51) | 0.89 | (0.78,1.02) | 1.59 | (1.39,1.82) | 3.09 | (2.69,3.55) |
| **Inland Midwest** | 1289 | 0.94 | (0.88,1.01) | 0.65 | (0.61,0.68) | 0.36 | (0.3,0.42) | 0.68 | (0.63,0.73) | 1.19 | (1.12,1.27) | 1.92 | (1.71,2.15) |
| **Inland South** | 2449 | 1.11 | (0.98,1.23) | 0.71 | (0.64,0.78) | 0.39 | (0.34,0.45) | 0.7 | (0.63,0.77) | 1.29 | (1.16,1.43) | 2.3 | (1.98,2.67) |
| **Inland Northeast** | 729 | 1.56 | (1.36,1.75) | 0.88 | (0.74,1.03) | 0.4 | (0.25,0.66) | 0.9 | (0.73,1.10) | 1.78 | (1.49,2.13) | 3.42 | (3.05,3.83) |
|  |  |  |  |  |  |  |  |  |  |  |  |  |  |
| **Coastal Status** | |  |  |  |  |  |  |  |  |  |  |  |  |
| **Coastal** | 4477 | 1.92 | (1.76,2.08) | 1.12 | (1.05,1.20) | 0.56 | (0.51,0.61) | 1.09 | (1.00,1.19) | 2.17 | (1.98,2.37) | 4.3 | (3.86,4.78) |
| **Non-coastal** | 5704 | 1.18 | (1.09,1.26) | 0.74 | (0.70,0.78) | 0.39 | (0.37,0.42) | 0.73 | (0.68,0.78) | 1.39 | (1.30,1.48) | 2.5 | (2.27,2.75) |
|  |  |  |  |  |  |  |  |  |  |  |  |  |  |
| **Survey Cycle** |  |  |  |  |  |  |  |  |  |  |  |  |  |
| **1999-2000** | 1640 | 1.96 | (1.46, 2.46) | 1.01 | (0.83, 1.25) | 0.41 | (0.32, 0.53) | 0.94 | (0.76, 1.17) | 2.03 | (1.47, 2.8) | 4.73 | (3.56, 6.28) |
| **2001-2002** | 1815 | 1.43 | (1.22, 1.64) | 0.83 | (0.74, 0.93) | 0.38 | (0.32, 0.45) | 0.79 | (0.71, 0.87) | 1.6 | (1.40, 1.84) | 3.02 | (2.66, 3.43) |
| **2003-2004** | 1617 | 1.35 | (1.15, 1.55) | 0.82 | (0.71, 0.94) | 0.39 | (0.31, 0.48) | 0.76 | (0.66, 0.88) | 1.51 | (1.30, 1.76) | 3.04 | (2.48, 3.72) |
| **2005-2006** | 1804 | 1.45 | (1.25, 1.64) | 0.92 | (0.82, 1.02) | 0.47 | (0.40, 0.55) | 0.89 | (0.8, 1.00) | 1.64 | (1.41, 1.92) | 3.11 | (2.77, 3.50) |
| **2007-2008** | 1510 | 1.25 | (1.06, 1.43) | 0.79 | (0.70, 0.88) | 0.41 | (0.37, 0.46) | 0.76 | (0.68, 0.85) | 1.42 | (1.23, 1.64) | 2.72 | (2.2, 3.36) |
| **2009-2010** | 1798 | 1.39 | (1.25, 1.54) | 0.88 | (0.80, 0.97) | 0.44 | (0.38, 0.52) | 0.83 | (0.73, 0.94) | 1.63 | (1.43, 1.86) | 3.12 | (2.85, 3.40) |

*unweighted sample size and weighted means.

Table 2. Difference in mean blood mercury levels, for women 16-49 years of age, by survey cycle NHANES 1999-2010.

| **Survey Cycle** | **Difference between Means** | **95% Confidence Limits** | |
| --- | --- | --- | --- |
| **1999-2000** | reference | --- | --- |
| **2001-2002** | -0.53 | -1.03 | -0.03 |
| **2003-2004** | -0.61 | -1.11 | -0.11 |
| **2005-2006** | -0.51 | -1.01 | -0.02 |
| **2007-2008** | -0.72 | -1.21 | -0.22 |
| **2009-2010** | -0.57 | -1.04 | -0.08 |

*F=18.23, <0.01

Table 3. Total fish consumption and type of fish consumed, by selected demographic variables among women 16-49 years of age participating in NHANES during 1999-2010.

| **Time** | **Total Fish Consumption**  **per month** | **Marine** | **Freshwater** | **Tuna** | **Shark/swordfish** | **Shellfish** |  |
| --- | --- | --- | --- | --- | --- | --- | --- |
| **1999-2000** | 3.6 ± 5.3 | 1.0 ± 2.2  (28.3) | 0.2 ± 0.9  (7.0) | 0.9 ± 2.2  (25.1) | 0.02 ± 0.19  (0.55) | 1.4 ± 3.0  (39.5) |  |
| **2001-2002** | 3.9 ± 5.5 | 1.2 ± 2.2  (30.8) | 0.3 ± 1.2  (7.2) | 0.8 ± 1.9  (20.3) | 0.02 ± 0.17  (0.41) | 1.6 ± 3.0  (41.3) |  |
| **2003-2004** | 4.2 ± 5.4 | 1.3 ± 2.6  (31.3) | 0.3 ± 0.9  (6.5) | 0.9 ± 1.9  (20.8) | 0.01 ± 0.11  (0.2) | 1.7 ± 3.0  (41.3) |  |
| **2005-2006** | 4.0 ± 5.2 | 1.2 ± 2.4  (30.2) | 0.3 ± 1.1  (8.7) | 0.9 ± 1.9  (21.9) | 0.01 ± 0.16  (0.26) | 1.6 ± 3.1  (38.9) |  |
| **2007-2008** | 4.1 ± 6.1 | 1.1 ± 3.3  (27.3) | 0.4 ± 1.1  (8.8) | 1.1 ± 2.4  (26.3) | 0.01 ± 0.14  (0.35) | 1.5 ± 2.8  (37.3) |  |
| **2009-2010** | 4.6 ± 6.0 | 1.6 ± 2.8  (34) | 0.2 ± 1.1  (5.1) | 0.8 ± 1.8  (18) | 0.01 ± 0.21  (0.24) | 1.9 ± 3.3  (42.3) |  |
| **Income** | | | | | | | |
| **<$20,000** | 3.5 ± 5.8 | 1.0 ± 3.0  (29.6) | 0.3 ± 1.3  (9.2) | 0.7 ± 1.8  (20.8) | 0.01 ± 0.09  (0.14) | 1.4 ± 3.0  (40.2) |  |
| **$20,000-$44,999** | 3.9 ± 5.3 | 1.1 ± 2.3  (28.7) | 0.3 ± 1.0  (7.4) | 0.9 ± 2.1  (23.1) | 0.01 ± 0.11  (0.2) | 1.6 ± 3.0  (40.6) |  |
| **$45,000-$74,999** | 4.3 ± 5.5 | 1.4 ± 2.6  (31.6) | 0.3 ± 1.0  (7.0) | 0.9 ± 1.9  (21.9) | 0.01 ± 0.13  (0.31) | 1.7 ± 3.0  (39.2) |  |
| **$75,000+** | 5.0 ± 5.9 | 1.6 ± 2.7  (32.1) | 0.2 ± 0.8  (4.9) | 1.1 ± 2.4  (22.7) | 0.03 ± 0.27  (0.6) | 2.0 ± 3.1  (39.7) |  |
| **Race/Ethnicity** | | | | | | | |
| **Non-Hispanic White** | 4.6 ± 6.3 | 1.3 ± 2.4  (27.7) | 0.2 ± 0.8  (4.2) | 1.2 ± 2.4  (25.5) | 0.02 ± 0.21  (0.43) | 1.6 ± 2.8  (34.1) |  |
| **Mexican American** | 3.0 ± 4.1 | 0.7 ± 1.7  (24.7) | 0.2 ± 0.7  (5.9) | 0.7 ± 1.6  (23.7) | 0.01 ± 0.11  (0.29) | 1.4 ± 2.4  (45.4) |  |
| **Other Hispanic** | 4.1 ± 5.7 | 1.2 ± 2.1  (30.2) | 0.2 ± 0.7  (3.8) | 1.0 ± 2.2  (23.6) | 0.01 ± 0.11  (0.23) | 1.7 ± 3.0  (42.2) |  |
| **Other** | 6.4 ± 8.8 | 2.5 ± 6.0  (39.3) | 0.5 ± 1.6  (7.3) | 0.8 ± 1.6  (12.4) | 0.02 ± 0.23  (0.30) | 2.6 ± 3.9  (40.6) |  |
| **Non-Hispanic Black** | 4.6 ± 6.3 | 1.5 ± 2.9  (32.8) | 0.6 ± 1.6  (13.3) | 0.6 ± 1.7  (13.2) | 0.01 ± 0.13  (0.14) | 1.9 ± 3.7  (40.6) |  |
| **Age** | | | | | | | |
| **16-19** | 2.5 ± 3.8 | 0.6 ± 1.6  (25.4) | 0.2 ± 0.9  (7.7) | 0.6 ± 1.5  (22.0) | 0.01 ± 0.12  (0.22) | 1.1 ± 2.2  (44.7) |  |
| **20-29** | 3.8 ± 5.0 | 1.1 ± 2.2  (28.4) | 0.2 ± 0.9  (6.2) | 0.9 ± 2.1  (23.8) | 0.01 ± 0.13  (0.25) | 1.6 ± 2.8  (41.3) |  |
| **30-39** | 4.8 ± 6.3 | 1.5 ± 2.7  (30.8) | 0.3 ± 1.0  (6.7) | 1.0 ± 2.2  (21.1) | 0.02 ± 0.23  (0.44) | 2.0 ± 3.7  (41.0) |  |
| **40-49** | 5.1 ± 6.5 | 1.8 ± 3.6  (34.4) | 0.4 ± 1.4  (8.1) | 1.1 ± 2.1  (21.1) | 0.02 ± 0.16  (0.32) | 1.8 ± 3.2  (36.0) |  |
| **Geographic Region** | | | | | | | |
| **Inland Midwest** | 3.8± 0.1 | 1.3 ± 0.1 (34.2) | 0.3 ± 0.0 (7.9) | 1.0 ± 0.1 (26.3) | 0.003 ± 0.0 (0.07) | 1.3 ± 0.1 (34.2) |  |
| **Great Lakes** | 4.1 ± 0.2 | 1.1 ± 0.1 (26.9) | 0.5 ± 0.0  (12.2) | 1.1 ± 0.1 (26.9) | 0.01 ± 0.0 (0.24) | 1.3 ± 0.1 (31.7) |  |
| **Inland South** | 4.2 ± 0.2 | 1.2 ± 0.1 (28.6) | 0.4 ± 0.0 (9.5) | 1.0 ± 0.1 (23.8) | 0.01 ± 0.0  (0.23) | 1.6 ± 0.1 (38.1) |  |
| **Inland Northeast** | 4.2± 0.2 | 1.3 ± 0.0 (30.1) | 0.1 ± 0.0  (2.3) | 1.2 ± 0.1  (28.6) | 0.02 ± 0.0  (0.47) | 1.7 ± 0.1  (40.5) |  |
| **Inland West** | 4.7 ± 0.2 | 1.7 ± 0.1 | 0.2 ± 0.0 | 1.3 ± 0.1 | 0.01 ± 0.0 | 1.6 ± 0.1 |  |
| **Pacific Coast** | 5.5 ± 0.2 | 2.0 ± 0.1  (36.4) | 0.2 ± 0.0  (3.6) | 1.1 ± 0.1 (20.0) | 0.03 ± 0.0  (0.54) | 2.2 ±0.1 (40.0) |  |
| **Gulf of Mexico** | 5.5± 0.1 | 1.4 ± 0.0  (25.5) | 0.5 ± 0.0  (9.1) | 0.9 ± 0.0  (16.4) | 0.01 ± 0.0  (0.18) | 2.8 ± 0.0  (50.9) |  |
| **Atlantic Coast** | 5.8 ± 0.1 | 1.8 ± 0.1 (31.0) | 0.2 ± 0.0 (3.4) | 1.3 ± 0.0  (22.4) | 0.04 ± 0.0  (0.69) | 2.5 ± 0.1 (43.1) |  |
